# Bioinformatic Analysis of Neuroimmune Mechanism of Neuropathic Pain

**DOI:** 10.1155/2020/4516349

**Published:** 2020-08-28

**Authors:** Hao Yu, Yang Liu, Chao Li, Jianhao Wang, Bo Yu, Qiang Wu, Ziqian Xiang, Shiqing Feng

**Affiliations:** ^1^Department of Orthopedics, Tianjin Medical University General Hospital, Tianjin, China; ^2^International Science and Technology Cooperation Base of Spinal Cord Injury, Tianjin Key Laboratory of Spine and Spinal Cord Injury, Department of Orthopedics, Tianjin Medical University General Hospital, Tianjin, China; ^3^Department of Medicine, Lincoln Medical Center, 234E 149th Street, Bronx, NY 10451, USA

## Abstract

**Background:**

Neuropathic pain (NP) is a devastating complication following nerve injury, and it can be alleviated by regulating neuroimmune direction. We aimed to explore the neuroimmune mechanism and identify some new diagnostic or therapeutic targets for NP treatment via bioinformatic analysis.

**Methods:**

The microarray GSE18803 was downloaded and analyzed using R. The Venn diagram was drawn to find neuroimmune-related differentially expressed genes (DEGs) in neuropathic pain. Gene Ontology (GO), pathway enrichment, and protein-protein interaction (PPI) network were used to analyze DEGs, respectively. Besides, the identified hub genes were submitted to the DGIdb database to find relevant therapeutic drugs.

**Results:**

A total of 91 neuroimmune-related DEGs were identified. The results of GO and pathway enrichment analyses were closely related to immune and inflammatory responses. PPI analysis showed two important modules and 8 hub genes: *PTPRC*, *CD68*, *CTSS*, *RAC2*, *LAPTM5*, *FCGR3A*, *CD53*, and *HCK*. The drug-hub gene interaction network was constructed by Cytoscape, and it included 24 candidate drugs and 3 hub genes.

**Conclusion:**

The present study helps us better understand the neuroimmune mechanism of neuropathic pain and provides some novel insights on NP treatment, such as modulation of microglia polarization and targeting bone resorption. Besides, *CD68*, *CTSS*, *LAPTM5*, *FCGR3A*, and *CD53* may be used as early diagnostic biomarkers and the gene HCK can be a therapeutic target.

## 1. Introduction

Neuropathic pain (NP) is an abnormal painful condition caused by a lesion or disease affecting the somatosensory nervous system [1]. The pain is usually described to be persistent and unbearable and seriously compromises the patient's quality of life [2]. Interestingly, nerve injury that happened to children below 5-6 years old seldom triggers neuropathic pain [3, 4]. Working on this observation, some researchers found that neuroimmune regulation was involved in the pathological process of neuropathic pain [5, 6]. Some studies further found the activation of glial cells and the production of a large number of cytokines that happened in neuroimmune microenvironment after neuropathic pain. However, the underlying mechanism about neuroimmune in regulating pain is still unclear.

Considering the importance of neuroimmune activation for neuropathic pain, several therapies targeting those mechanisms have been developed and proved to be effective [7]. For example, the inhibition of TNF-*α*, a pro-inflammatory cytokine, could reduce the hyperalgesia induced by diabetic neuropathy [8]. Besides, some researchers found that the Toll-like receptor is responsible for gliosis after nerve injury and knockout of TLR2 could attenuate nociceptive hypersensitivity in mice [9]. Some other treatments such as intrathecal injection of IL-10 and gene transfer of IL-4 using a viral-mediated vector have similar results [10]. Therefore, it is valuable to find some new therapeutic targets especially the upstream regulating genes.

In recent decades, the advancement of microarray technology and bioinformatics enable researchers to study the biological mechanism more comprehensively and make it more effective to identify key therapeutic and prognostic molecules. In this study, we downloaded GSE18803 from the Gene Expression Omnibus (GEO) database datasets. Subsequently, we identified a series of differentially expressed genes using R software. We further performed a series of comprehensive bioinformatic analysis on those differentially expressed genes. Our study is aimed at providing new therapeutic targets for neuropathic pain and helping to understand the underlying neuroimmune mechanism of neuropathic pain.

## 2. Material and Methods

### 2.1. Microarray Data

The microarray dataset GSE18803 was retrieved from GEO (https://www.ncbi.nlm.nih.gov/geo/). It was deposited by Costigan et al. and contained 24 samples (adult rats, 6 in the surgery group vs. 6 in the sham group; neonate rats, 6 in the surgery group vs. 6 in the sham group) [11]. In the surgery group, the adult (8-12 weeks) and neonate (10 days) rats were performed spared nerve injury (SNI) where the tibial and common peroneal nerve was ligated with a 5.0 silk and transected distally, removing 2-4 mm of distal nerve stump while leaving the sural nerve intact. In the sham operation group, the sciatic nerve was exposed but not ligated. 7 days post-SNI, the L4-L5 lumbar dorsal horns ipsilateral to the injury were collected and processed for total RNA extraction. The platform for gene expression detection was GPL341 (Affymetrix Rat Expression 230A Array).

### 2.2. Data Preprocessing and DEG Identification

Firstly, the raw CEL file was downloaded and divided into two separate documents—the adult and neonate groups—uploaded to R software (v 3.6.2). RMA (Robust multiarray average) method in the Affy package was used to perform normalization and background correction. KNN (*K*-nearest neighbors) method in the impute package was used to supplement the missing value. Finally, a total of 15923 probes were obtained both in the adult and neonate gene expression matrices. After that, the probe ID was converted into an official gene symbol based on the GPL341 annotation file downloaded from the Affymetrix official website.

Bayes test in the limma package was used to identify DEGs between the SNI and sham injury groups in each age group. The DEGs with ∣LogFC | >0.5 and *P* value < 0.05 were considered to be significant. After that, Venn analysis was performed between the two lists of DEGs.

### 2.3. Function and Pathway Enrichment Analyses

GO provides a specific language to describe the attributes of the selected genes in three main domains which are cellular component (CC), molecular function (MF), and biological process (BP) [12]. GO function enrichment analysis was performed by an online web tool provided in the DAVID database (https://david.ncifcrf.gov/). The Kyoto Encyclopedia of Genes and Genomes (KEGG) database stores relevant pathway information which can help us understand cells, organisms, and ecosystems at the molecular level [13]. KEGG pathway enrichment analysis was performed by another online web tool provided in the KOBAS database (http://kobas.cbi.pku.edu.cn/). TheDEGs only found in the adult groups were uploaded in the database and performed GO and KEGG pathway enrichment analyses. *P* value < 0.05 was set as a cutoff.

### 2.4. PPI Network Construction and Analysis

The gene list was uploaded to the Multiple Protein web tool in the STRING database (https://string-db.org/) and a combined score > 0.4 was set as the cutoff. The TSV file was downloaded and submitted to Cytoscape software, an open-source software platform for visualizing complex networks, to visualize the PPI network. 12 centrality mathematical calculation methods in cytoHubba, a java plugin in Cytoscape, were all applied to screen hub genes by analyzing the topology of the constructed protein-protein interaction networks. The genes with counts over 6 were chosen as the hub gene.

Molecular Complex Detection (MCODE), an app for cluster analysis in Cytoscape, was used to find important functional modules in the constructed PPI network. The genes contained in the two selected modules were performed pathway enrichment analysis in the METASCAPE database (https://metascape.org), respectively.

### 2.5. Related Drug Prediction

The 8 hub genes were served as promising targets for therapeutic drug retrieval in the DGIdb database (http://dgidb.genome.wustl.edu/), a web source that helps discover drug-gene interactions. The preset filters were set to be “FDA Approved” and “Immunotherapies,” while the advanced filters were set to include 20 databases, 41 gene categories, and 51 gene interaction types. The results were visualized in Cytoscape.

## 3. Result

### 3.1. Identification of DEGs

**Figure 1:**
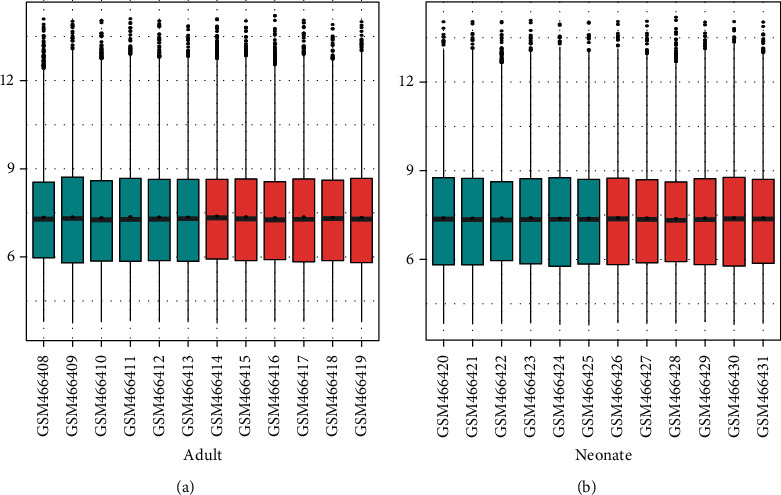
Box plot of expression value of two age groups. (a) The expression value of the adult rat group. (b) The expression value of the neonate rat group. The horizontal axis represents the sample name, while the vertical axis represents the expression value after normalization. The blue represents the sham group, while the red represents the spared nerve injury (SNI) group. The black line in the box represents the median of value, which can stand for the degree of normalization. They were all in the same line, indicating the normalization was effective.

**Figure 2:**
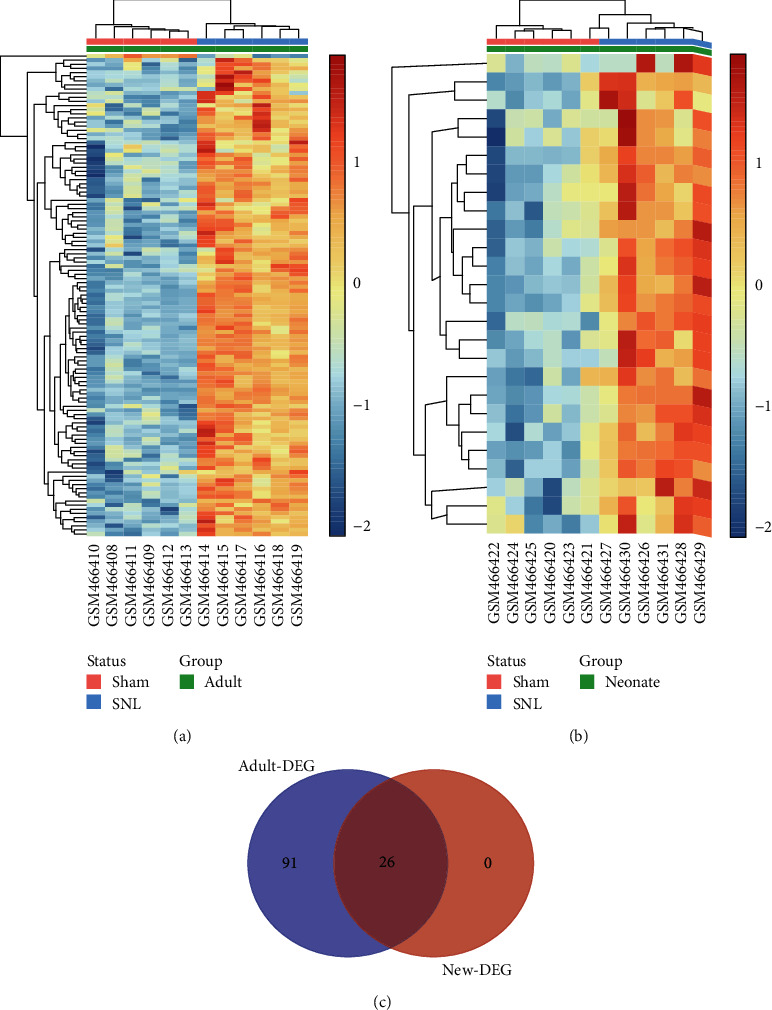
The heat map of differentially expressed genes (DEGs) in the adult rat group (a). The heat map of DEGs in the neonate rat group (b). The horizontal axis represents the name of each sample, while the left vertical axis represents the degree of gene clustering. Red stands for the upregulated genes, while blue stands for the downregulated genes (a, b). Venn diagram of DEGs. The 26 DEGs in the neonate group were all included in the adult group. 91 DEGs were only expressed in the adult group (c).

To ensure the reliability of the analysis results, we firstly performed data background correction and normalization (Figures 1(a) and 1(b)). Then, we identified 117 DEGs in the adult rat group (116 upregulated and 1 downregulated) and 26 DEGs (all upregulated) in the neonate rat group through DEG analysis using R. The results were visualized through a heat map (Figures 2(a) and 2(b)). Venn diagram analysis demonstrated that the 24 DEGs in the neonate groups were all included in the adult group DEG list (Figure 2(c)). We aimed to explore the underlying neuroimmune mechanism of neuropathic pain and find several valuable pain biomarkers, while the neonate rats did not develop nerve pain after nerve injury [14, 15]. Therefore, we mainly focused on the DEGs dysregulated only in the adult groups.

### 3.2. GO and KEGG Analyses

To describe the attributes of selected DEGs, we submitted the gene list to the DAVID database and performed GO function enrichment analysis. We have found 148 GO terms with the *P* value < 0.05 (100 BP items, 28 CC items, and 20 MF items). The top 20 GO terms, ranked by *P* value, were shown in Table 1. Most of the enriched terms were about BP. We further found that many BP terms were closely related to the inflammatory and immune processes, such as inflammatory response, cellular response to lipopolysaccharide, B cell receptor signaling pathway, regulation of B cell, and positive regulation of T cell activation.

To further investigate the functions of the DEGs, we did KEGG pathway analysis in the KOBAS database. The top 10 pathways were shown in Table 2. The result was similar to the GO analysis. Several most significantly enriched pathways were involved in inflammatory and immune responses, such as Fc gamma R-mediated phagocytosis, natural killer cell-mediated cytotoxicity, chemokine signaling pathway, and Yersinia infection.

### 3.3. PPI Network Analysis

**Figure 3:**
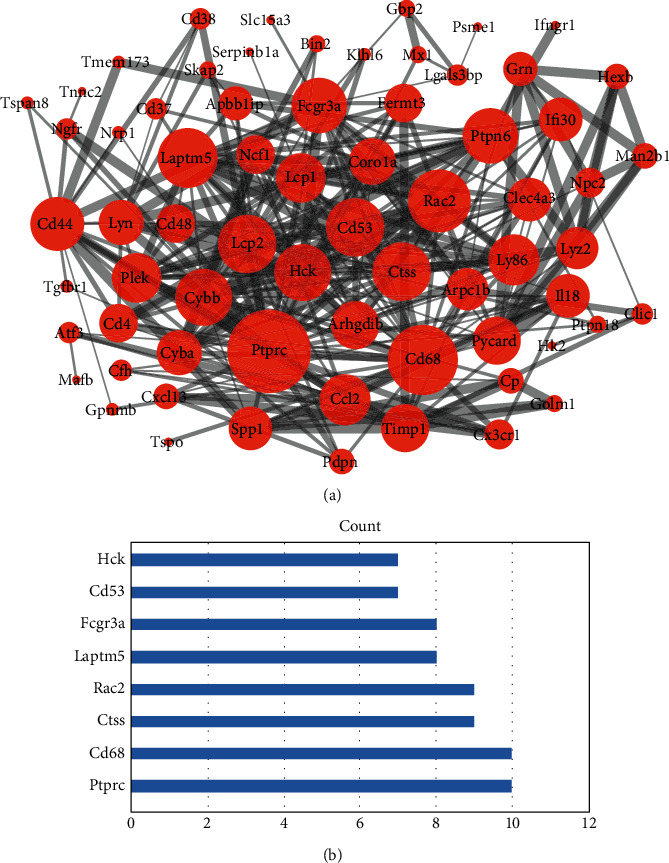
Protein-protein interaction network of 91 neuroimmune-related genes. The size of each protein is determined by its connection degree to other proteins. The width of each edge is determined by the combined score between the related two proteins (a). The 8 hub genes screened by 12 centrality mathematical calculation methods (b).

Based on the information from the STRING database, the current established PPI network contained 68 nodes and 299 protein pairs (Figure 3(a)). To identify the hub gene in the PPI network, we applied every calculation method in the cytoHubba plugin. The genes with number > 6 were selected as the hub gene, including protein tyrosine phosphatase receptor type C (*Ptprc*), Cd68 molecule (*Cd68*), cathepsin S (*Ctss*), Rac family small GTPase 2 (*Rac2*), lysosomal protein transmembrane 5 (*Laptm5*), Fc fragment of IgG receptor IIIa (*Fcgr3a*), CD53 molecule (*Cd53*), protooncogene, and hematopoietic cell kinase (*Hck*) (Figure 3(b)).

**Figure 4:**
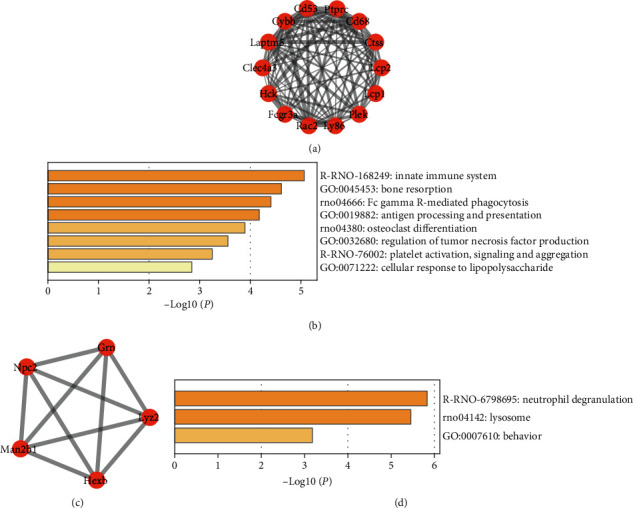
The first module (a) and the pathway enrichment analysis of this module (b). The second module (c) and the pathway enrichment analysis of this module (d).

To further analyze the PPI network, we found two important clusters using the MCODE plugin in Cytoscape and performed pathway enrichment in the METASCAPE database (Figures 4(a)–4(d)). The first cluster was mainly enriched in the innate immune system, bone resorption, Fc gamma R-mediated phagocytosis, and antigen processing and presentation. The second cluster was enriched in neutrophil degranulation and lysosome.

### 3.4. Related Drug Prediction

**Figure 5:**
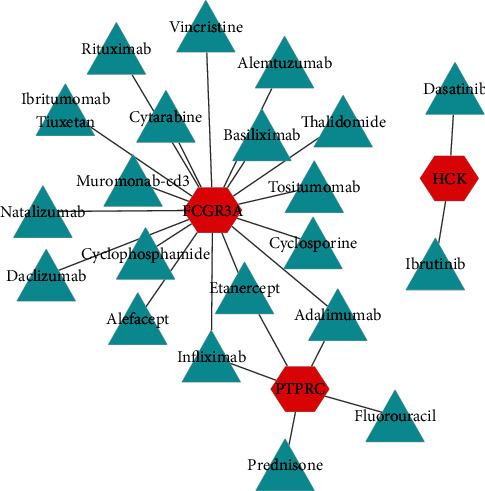
The drug-hub gene interaction network. The red nodes represent the hub genes, while the blue nodes represent the relevant drugs. A total of 3 hub genes and 24 drugs were included in the network.

Using the DGIdb database, the potential drugs targeting the selected genes were obtained. The results showed that a total of 24 candidate drugs included *PTPRC* (prednisone, fluorouracil, adalimumab, infliximab, and etanercept), *FCGR3A* (rituximab, muromonab-Cd3, alefacept, natalizumab, daclizumab, Ibritumomab tiuxetan, etanercept, tositumomab, adalimumab, basiliximab, ciclosporin, alemtuzumab, thalidomide, cytarabine, cyclophosphamide, vincristine, and infliximab), and *HCK* (dasatinib and ibrutinib), as shown in Figure 5. Further use of literature retrieval found that prednisone, adalimumab, infliximab, etanercept, alemtuzumab, and thalidomide have been clinically used for NP treatment. Four drugs (rituximab, natalizumab, daclizumab, and ibrutinib) have a role in neuropathy.

## 4. Discussion

The nerve injury occurred to neonate rats seldom triggers neuropathic pain, while in adults it does [16, 17]. Research on DEGs between the neonate and adult rats after SNI might help us better understand the underlying mechanism of neuropathic pain. In the current study, we downloaded the GSE18803 dataset from GEO and identified 91 DEGs particularly expressed in adult rats after SNI. The 91 DEGs were then used to perform GO, pathway, and PPI network analyses. We also determined several hub genes and constructed the hub gene-drug network. Our results might provide some novel insights in neuropathic pain treatment.

Previous studies demonstrated that the activation of neuroimmune mechanisms after nerve injury was one of the main reasons to cause neuropathic pain [18, 19]. The results of GO and pathway enrichment analyses have shown similar trends, since many enriched terms or pathways were immune related. What is more, according to the results of enrichment analysis, we hypothesize that the infiltration of immune cells, including B cells, T cells, and natural killer cells, was closely related to the development of neuropathic pain.

We identified 8 hub genes in the PPI network: *PTPRC*, *CD68*, *CTSS*, *RAC2*, *LAPTM5*, *FCGR3A*, *CD53*, and *HCK*. Interestingly, five among them (*CD68*, *CTSS*, *LAPTM5*, *FCGR3A*, and *CD53*) were involved in microglia activation. *CD68* is a widely used biomarker for activated macrophages or microglia. Its overexpression represents the functional phenotype of macrophages/microglia changing to proinflammatory M1 cells which directly induce neuropathic pain [20, 21]. Both *CTSS* and *LAPTM5* were lysosome-associated proteins in the microglia. The critical role of *CTSS* played in maintaining neuropathic pain has been proved, while the function of *LAPTM5* in neuropathic pain needs further elucidation [22–24]. *FGCR3A* was also identified as a crucial gene in neuropathic pain before [25]. Besides, the expression of *FGCR3A* was positively correlated with microglia phagocytic capacity [26]. *CD53*, the cell surface adhesion molecule, was overexpressed in the microglia after nerve injury and might mediate microglial migration [27]. Considering the early response of the microglia in the generation of neuropathic pain, the five identified genes have the potential to be the early diagnostic biomarkers of neuropathic pain [28].

Multiple studies have suggested that activated microglia were a critical contributor to the initiation and development of neuropathic pain [29, 30]. However, there are two different states of microglia activation, the proinflammatory M1 and anti-inflammatory M2. As opposed to M1 microglia which releases proinflammatory or neurotoxic factors, inducing NP, M2 microglia release anti-inflammatory factors, dampening inflammation and subsequently alleviating NP. A recent study showed that although both M1 and M2 microglia are activated after nerve injury, the microglia skewed towards M1 phenotype as neuralgia develops [31]. This might explain why inhibiting microglia activation could alleviate NP, regardless of its phenotype [32, 33]. What is more, some researchers found microglia polarization to the M2 phenotype could reduce neuropathic pain [34, 35]. Therefore, modulation of microglia polarization, inhibiting proinflammatory M1 microglia reaction while simultaneously promoting M2 microglia anti-inflammatory effect could be another treatment for neuropathic pain.

*HCK* belongs to the Src protein family (SFK). SFKs extensively participate in multiple signal transduction pathways such as cell growth, migration, and apoptosis. Recently, some researchers have found that the spinal cord injury could result in SFK activation in the microglia, and administration of the SFK inhibitor could attenuate mechanical hypersensitivity following nerve injury [36]. Specifically, as a member of SFKs, *HCK* was also found upregulated during microglia activation [37]. Previous studies demonstrated that *HCK* could mediate TLR4-induced proinflammatory mediator production via AP-1 [38]. TLR4, a specific pattern recognition receptor expressed on the microglia surface, was reported to play an important role during the pathological process of neuropathic pain [39, 40]. Therefore, it is reasonable to speculate that *HCK* is associated with the generation of neuropathic pain. The overexpressed *HCK* might activate the microglia and increase the production of proinflammatory cytokines via the TLR/AP-1 pathway, subsequently inducing neuropathic pain. What is more, the gene therapy targeting HCK might attenuate the severe neuropathic pain.

The pathway enrichment analysis of the top 2 modules showed those genes were mainly enriched in the innate immune system, bone resorption, Fc gamma R-mediated phagocytosis, antigen processing and presentation, neutrophil degranulation, and lysosome. It is reasonable that most of the enriched terms were immune related. Besides, bone resorption attracted our attention. Bisphosphonates, the compounds commonly used for osteoporosis treatment, have recently been found to also alleviate neuropathic pain [41]. Bisphosphonate compounds can inhibit bone resorption by inhibiting osteoclast activation and inducing osteoclast apoptosis. The osteoclasts and microglia both belong to the mononuclear macrophage family, and they are both differentiated from hematopoietic stem cells [42]. The bisphosphonate compounds targeting receptors on osteoclasts such as CD45 were also expressed on the microglia [43, 44]. Yao et al. reported alendronate, one of the bisphosphonate compounds, could inhibit CD45 downstream signaling pathways, attenuate microglial activation, and subsequently alleviate neuropathic pain [44]. Therefore, the studies on bone resorption can not only help treat osteoporosis, another serious complication following nerve injury, but also provide a novel therapeutic target for neuropathic pain.

The DGIdb database makes it convenient to screen therapeutic agents that might relieve neuralgia. Mounting epidemiological studies demonstrated TNF-*α* blockade dampening inflammation and subsequently alleviating NP. Adalimumab, infliximab, and etanercept are all TNF-*α* inhibitors, and they have been clinically used for NP treatment [45–47]. However, during the treatment, neutralizing antibodies would develop to adalimumab and infliximab, attenuating treatment efficacy, while etanercept would not [48]. Thus, etanercept could be in consideration for some refractory NP treatment. Thalidomide is another promising approach for NP treatment. Some animal studies have demonstrated that oral or intraperitoneal injection of thalidomide can not only reduce NP responses but also mediate depressive-like behaviors associated with NP [49, 50]. The others, including natalizumab, rituximab, daclizumab, and ibrutinib, have not been reported as NP therapeutic drugs. However, these drugs are commonly used in some neurological diseases, including multiple mononeuropathy (MM), pediatric multiple sclerosis (PMS), and paraneoplastic disorders of the peripheral nervous system, of which NP is a frequent complication [51–53]. Therefore, the treatment efficacies of these drugs in NP deserve further study.

Several limitations should be acknowledged in our study. First, the sample size was relatively small. Besides, the results were all based on bioinformatic analysis. The experimental and clinical verifications are lacking. In the future, we will perform some more in-depth study around the screened hub genes and drugs.

## 5. Conclusion

Taken together, the present study deepens our knowledge about the neuroimmune mechanism of neuropathic pain. The study could instruct the treatment of neuropathic pain, such as modulation of microglia polarization and targeting bone resorption. We also identified several hub genes and predicted relevant therapeutic drugs. Among them, *CD68*, *CTSS*, *LAPTM5*, *FCGR3A*, and *CD53* may be used as the early diagnostic biomarkers while the gene HCK can be a therapeutic target.

## Figures and Tables

**Table 1:** The top 20 most significant GO terms.

Category	Term	Count	*P* value
BP	GO:0006954~inflammatory response	14	2.42*E* − 09
BP	GO:0071222~cellular response to lipopolysaccharide	10	1.41*E* − 07
BP	GO:0050853~B cell receptor signaling pathway	6	3.13*E* − 06
BP	GO:0045577~regulation of B cell differentiation	4	1.38*E* − 05
BP	GO:0051279~regulation of release of sequestered calcium ion into cytosol	4	5.14*E* − 05
BP	GO:0050870~positive regulation of T cell activation	4	9.12*E* − 05
BP	GO:0071346~cellular response to interferon-gamma	5	2.38*E* − 04
BP	GO:0071407~cellular response to organic cyclic compound	6	3.52*E* − 04
BP	GO:0051607~defense response to virus	6	4.39*E* − 04
BP	GO:0045730~respiratory burst	3	6.68*E* − 04
BP	GO:0009611~response to wounding	5	6.71*E* − 04
BP	GO:0050665~hydrogen peroxide biosynthetic process	3	8.56*E* − 04
CC	GO:0070062~extracellular exosome	37	8.92*E* − 10
CC	GO:0005764~lysosome	10	2.39*E* − 06
CC	GO:0005615~extracellular space	21	2.70*E* − 06
CC	GO:0009986~cell surface	12	1.49*E* − 04
CC	GO:0005623~cell	6	2.58*E* − 04
CC	GO:0043020~NADPH oxidase complex	3	7.86*E* − 04
MF	GO:0008201~heparin binding	6	5.76*E* − 04
MF	GO:0016175~superoxide-generating NADPH oxidase activity	3	5.81*E* − 04

GO: Gene Ontology; BP: biological process; CC: cellular component; MF: molecular component; Count: enriched gene numbers in each term; NADPH: nicotinamide adenine dinucleotide phosphate.

**Table 2:** The top 10 most significant enriched KEGG pathways.

ID	Description	No. of genes	*P* value	Input
rno04666	Fc gamma R-mediated phagocytosis	7	6.20*E* − 09	Ncf1, Rac2, Arpc1b, Fcgr3a, Ptprc, Hck, Lyn
rno04142	Lysosome	7	4.29*E* − 08	Ctsh, Hexb, Laptm5, Man2b1, Cd68, Npc2, Ctss
rno05140	Leishmaniasis	6	4.72*E* − 08	Ncf1, Fcgr3a, Cybb, Cyba, Ifngr1, Ptpn6
rno04650	Natural killer cell-mediated cytotoxicity	6	2.24*E* − 07	Rac2, Cd48, Fcgr3a, Ifngr1, Lcp2, Ptpn6
rno04621	NOD-like receptor signaling pathway	7	3.77*E* − 07	Il18, Ccl2, Gbp2, Cybb, Cyba, Pycard, Tmem173
rno04060	Cytokine-cytokine receptor interaction	8	3.84*E* − 07	Il18, Ccl2, Tgfbr1, Cx3cr1, Cd4, Ifngr1, Cxcl13, Ngfr
rno04062	Chemokine signaling pathway	7	3.91*E* − 07	Ccl2, Ncf1, Rac2, Cx3cr1, Hck, Cxcl13, Lyn
rno05135	Yersinia infection	6	9.44*E* − 07	Il18, Ccl2, Rac2, Skap2, Pycard, Lcp2
rno04380	Osteoclast differentiation	6	9.86*E* − 07	Ncf1, Fcgr3a, Tgfbr1, Cyba, Ifngr1, Lcp2
rno04145	Phagosome	6	1.07*E* − 05	Ncf1, Cyba, Fcgr3a, Cybb, Coro1a, Ctss

KEGG: Kyoto Encyclopedia of Genes and Genomes; NOD: nucleotide-binding oligomerization domain.

## Data Availability

The data used to support the findings of the study are included in this article.
